# Liquid phase selective oxidation of veratryl alcohol to veratraldehyde using pure and Mg-doped copper chromite catalysts

**DOI:** 10.1039/d4ra00846d

**Published:** 2024-06-05

**Authors:** S. Jagadeesan, V. Prathipa, C. Ragupathi, G. Ramalingam, S. Narayanan, P. Tamizhdurai, A. Rajendran, Krishna Kumar Yadav, Ghadah Shukri Albakri, Mohamed Abbas, Maha Awjan Alreshidi

**Affiliations:** a Department of Physics, Srimad Andavan Arts and Science College (Autonomous) (Affiliated to Bharathidasan University, Tiruchirappalli) Tiruchirappalli-620005 Tamilnadu India; b Department of Chemistry, PSNA College of Engineering Technology Dindigul Tamilnadu India; c Department of Chemistry, Sriram College of Arts and Science Perumalpattu Tiruvallur 602024 Tamilnadu India; d Department of Nanoscience and Technology, Alagappa University Karaikudi 630003 India; e Department of Chemistry, Dwaraka Doss Goverdhan Doss Vaishnav College (Autonomous) (Affiliated to the University of Madras) India tamizhvkt2010@gmail.com +91-9677146579; f Department of Chemistry, Sir Theagaraya College, (Affiliated to University of Madras) Chennai 600 021 Tamilnadu India; g Faculty of Science and Technology, Madhyanchal Professional University Ratibad Bhopal 462044 India; h Environmental and Atmospheric Sciences Research Group, Scientific Research Center, Al-Ayen University Thi-Qar Nasiriyah 64001 Iraq; i Department of Teaching and Learning, College of Education and Human Development, Princess Nourah Bint Abdulrahman University P. O. Box 84428 Riyadh 11671 Saudi Arabia; j Electrical Engineering Department, College of Engineering, King Khalid University Abha 61421 Saudi Arabia; k Department of Chemistry, University of Ha'il Ha'il 81441 Saudi Arabia

## Abstract

Mg-doped copper chromite (CuCr_2_O_4_) nanocomposites were synthesised through conventional technique. The pure and doped CuCr_2−*x*_Mg_*x*_ O_4_ (*x* = 0.00–0.1, 0.2 and 0.3%) nanocomposites were characterized in terms of their morphology, crystal structure, surface area and catalytic performance. The chemical composition of CuCr_2−*x*_Mgx O_4_ was confirmed *via* FT-IR. The formation of pure and doped catalysts was validated by XRD results. TEM/SEM confirmed the formation of CuCr_2−*x*_Mg_*x*_O_4_ nanoparticles. Mg-doped samples possess a high specific surface area compared to pure CuCr_2_O_4_. Thus, the effects of temperature, solvent, time, oxidant and the amount of catalyst on the oxidation of veratryl alcohol were reported. Furthermore, detailed mechanisms of the catalytic oxidation of veratryl alcohol as well as the reusability and stability of the nanomaterial were investigated. The resulting composites were shown to be effective heterogeneous catalysts for the oxidation of veratryl alcohol.

## Abstract 2

Mg-doped copper chromite (CuCr_2_O_4_) nanocomposites were synthesised through conventional technique.

## Graphical abstract

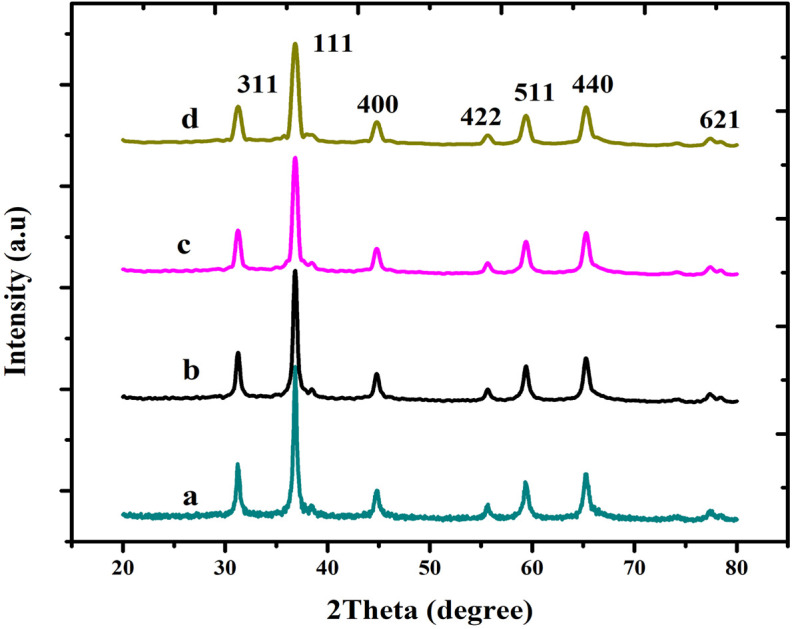

## 1. Introduction

In the field of heterogeneous catalysis, sunlight-activated materials are necessary. Because nanotechnology enables control over their size and morphology, there is a correlation between their increased efficiency and these developments. In the field of catalysis, ternary metal oxides with a spinel structure (AB_2_O_4_) have demonstrated exceptional properties. Thus, metal oxides were located around the octahedral (B site) and tetrahedral (A site) positions, and the quantities, type of oxides and the location of metal cations in the crystalline structure significantly affect the chemical–physical properties of chromite materials.^1,2^ At present, chromite materials with spinel structures are present with a face-centered lattice with the space group *Fd*3*m*, whereas Cr^3+^ cations are in octahedral positions and Cu^2+^ cations occupy tetrahedral positions.^3^ Chromium-based catalysts exhibit good resistance to atmospheric effects, high thermal stability, optical properties, resistance to chemical corrosion, photocatalysis, catalysis *etc.* Beyond that, it is interesting to see numerous applications for ceramic materials.^4^ The doping of divalent Mg^2+^ metal ions is primarily incorporated into A-site cations in the synthesized copper chromite materials. The stoichiometry of chemical compositions and the controlled crystallite size of chromite materials prepared via the chemical method were found to be extremely favorable for achieving good-quality products.^5^ Copper chromates were synthesized using various methods, such as sol–gel, ceramic,^6^ microwave and co-precipitation techniques.^7^ New chemical methods, such as hydrothermal, sol–gel/citrate,^8^ conventional (low-temperature method), and low-temperature solution synthesis methods, were also developed.^9^

Mg^2+^ doped chromite based catalysts were used in the selective oxidation reaction. Also, chromite based materials were more useful in industrial and technological applications.^10^ Selective oxidation of veratryl alcohol to carbonyl compounds (aldehyde to acid) is highly challenging.^11^ Selective oxidation of veratryl alcohol is employed to produce several industrially valuable chemicals, such as those associated with the food and fragrance industries; veratraldehyde is a highly valued chemical that is derived from crude oil and *via* the methylation of vanillin. It is interesting to note that the industry that utilizes veratraldehyde spends as much as 100 times more on bio-based veratraldehyde than on crude oil-based veratraldehyde.^12–14^ Herein, we synthesized citrate CuCr_2_O_4_ and Mg-doped CuCr_2_O_4_*via* a conventional combustion route, followed by their morphological and structural characterisation. The chemical–physical properties of the catalysts are characterised using multiple techniques. The resulting composites were shown to be effective heterogeneous catalysts for the oxidation of veratryl alcohol.

## 2. Experimental

At present, (CuCr_2_O_4_) copper chromite spinel oxides were (flow chart 1) prepared *via* the conventional combustion route. The following chemicals, such as citric acid C_6_H_8_O_7_ (used as fuel), chromium nitrate Cr (NO_3_)_3_·9H_2_O, copper nitrate Cu (NO_3_)_2_·6H_2_O and magnesium nitrate Mg (NO_3_)_2_·6H_2_O were used without purification. First stage (I): chromium nitrate 1 M solution and copper nitrate 0.5 M solution were prepared separately in 25 mL (DI) deionized water. Second stage (II): Cr and Cu solutions were mixed and stirred continuously for 1 h to obtain a homogenous solution. Third stage (III): An aqueous solution of 5 M citric acid was prepared in DI water, and the citrate to nitrate ratio was fixed at one. Then, the obtained solution (citrate) is dropped into the nitrate solution under continuous stirring, and the homogeneous solution is neutralized with the addition of ammonium hydroxide (NH_4_OH). Following this, different amounts (0.1%, 0.2%, and 0.3%) of magnesium nitrate hexahydrate (0.3 M) were introduced into the aforementioned solution. Fourth stage (IV): the overall homogenous solution was evaporated using a heating system; it was kept on a hot plate at 100 °C with intensive stirring for about 1–2 hours. Thus, a highly viscous gel was obtained. Fifth stage (V): the extremely viscous gel was then transferred to a muffle furnace, where self-ignition was initiated at an experimental temperature of 400 °C/5 hours. The samples (Table 1) of the (0.0) undoped CuCr_2_O_4_ and (0.1 to 0.3) from Mg-doped CuCr_2_O_4_ were labelled (a) and (b) to (d).

**Table 1:** Sample code and sample composition with their abbreviations

Sample code	Sample composition
a	CuCr_2_O_4_
b	Mg (0.1%) CuCr_2_O_4_
c	Mg (0.2%) CuCr_2_O_4_
d	Mg (0.3%) CuCr_2_O_4_

### 2.1. Instrumental

X-ray diffraction analysis was carried out using a Rigaku DMAX-3A powder diffractometer with CuKα radiation at a wavelength of 1.54 Å to investigate the crystalline phase and the various structural parameters of the prepared samples. These patterns of XRD were scanned in a 2*θ* range of 20° to 80° with a scanning step size of 0.05°. The morphology was characterized *via* field-emission scanning electron microscopy (FESEM) (SEM, QUANTA FEG 250, USA) and transmission electron microscopy (TEM). The specific surface areas (BET) of the products were tested using a Micromeritics ASAP 2020 apparatus. Fourier-transform infrared (FTIR) spectra were recorded using a thermo scientific Nicolet iS10 instrument in an *in situ* Harrick IR cell.

### 2.2. Catalytic test

Catalytic oxidation of alcohol was carried out on a batch reactor functioning under atmospheric conditions. 10 mmol of (CH_3_CN, DMF and CHCl_3_), 10 mmol of veratryl alcohol (at a molar ratio of H_2_O_2_/alcohol of 1 : 1), and 10 mmol of oxidant (TBHP/H_2_O_2_) was added along with 0.1 to 0.3 g of the pure and Mg-doped copper chromites, and the fillings were heated to 100 °C at different reaction times for 12 h at altered time intervals in a three-necked round bottom flask equipped with a reflux condenser and the thermometer.
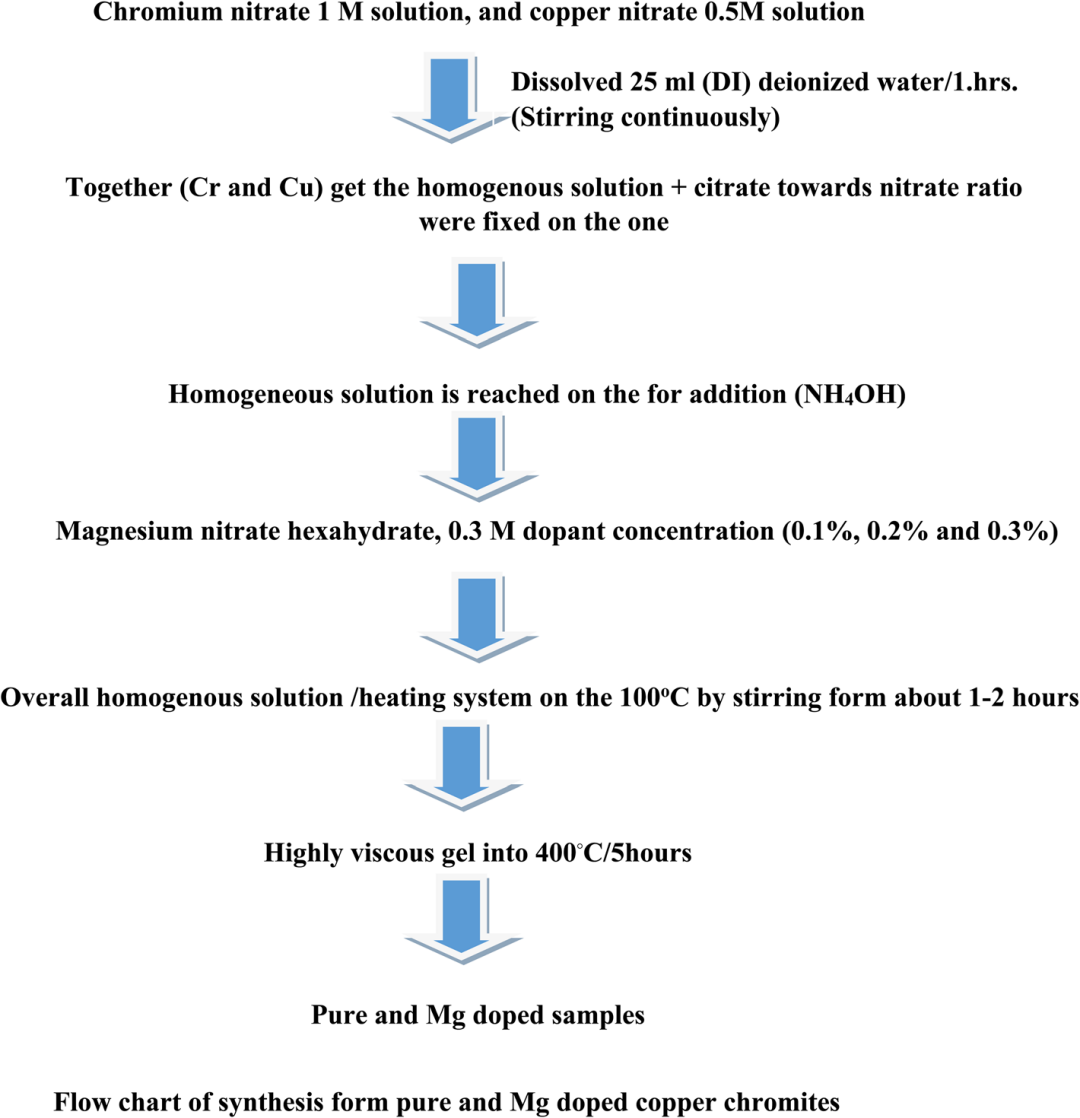


From these values, the overall conversion of the reactant veratryl alcohol and the selectivity of the product (3) was estimated.
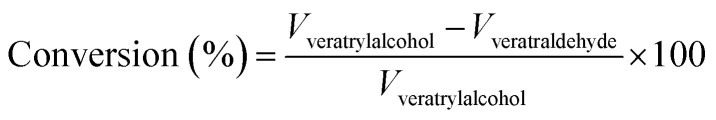

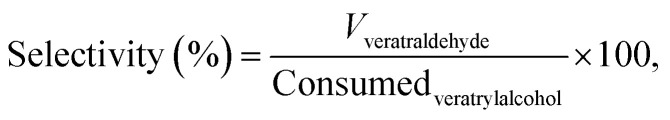

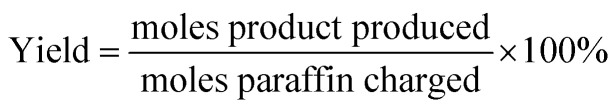


## 3. Results and discussion

### 3.1. XRD pattern of the Mg-doped CuCr_2_O_4_ nanocomposites

**Fig. 1:**
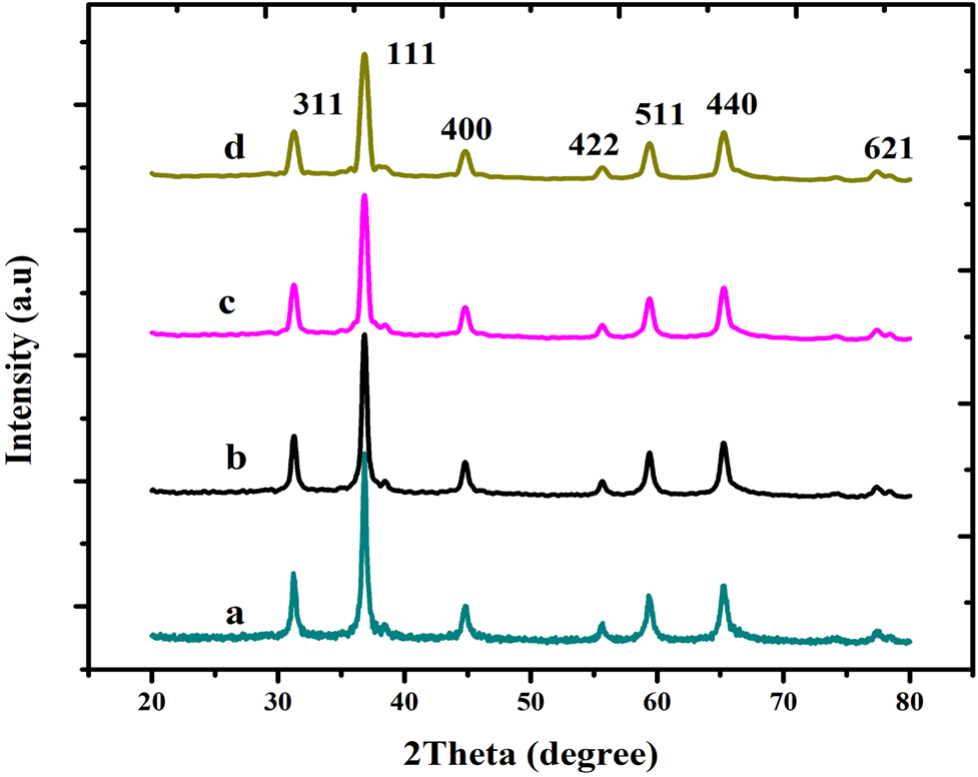
X-ray diffraction pattern of (a) CuCr_2_O_4_ and (b–d) Mg-doped CuCr_2_O_4_.

The XRD measurements were performed to determine the effectiveness of the production on the pure and Mg-doped CuCr_2_O_4_. As shown in Fig. 1, the obtained XRD peaks are indexed as undoped and Mg-CuCr_2_O_4_ with a cubic structure formation. As confirmed by a standard card (JCPDS No. 34-0424), these XRD peaks conform to undoped and Mg-CuCr_2_O_4_, matching well with the standard. As the amount of Mg doping increased from *x* = 0 to 0.1, 0.2, and 0.3%, the samples showed only copper chromite's phase, and the peak intensities were comparable with the pure CuCr_2_O_4_ sample. This indicates the integrity of the Mg dopant within the copper chromite crystal structure. XRD patterns ranging from 35.5° to 37.5° were assigned to the 111 plane, which has the highest intensity. Furthermore, the primary peaks at 2*θ*-values adhering to the (111) and (311) planes show a small change as the doping concentration of Mg quantity increases. The XRD peaks of the samples progressively changed between low and high angles, which correlated with the extension (Table 2) of the lattice parameters, cell volume (Å^3^) and *d* spacing values. Thus, ionic radii of Mg^2+^ = 86 pm, Cr^3+^ = 75.5 pm, and Cu^2+^ = 77 pm were compared. Mg-doped copper chromites have smaller crystallite sizes than pure copper chromites due to the substitution of the lattice ions by the Mg^2+^ ions and their entry into interstitial locations.^15,16^

**Table 2:** Crystallite size, lattice parameters and lattice strain of copper chromite and Mg-doped copper chromates

Samples	Crystallite size (nm)	Lattice parameters		Strain
		*a* = *b* (Å)	*c* (Å)	
a	43.12	5.9832	3.9810	3.234
b	38.45	5.9833	3.9811	3.091
c	31.45	5.9835	3.9833	2.981
d	29.98	5.9837	3.9835	2.567

The average crystallite size was determined using the Scherrer formula:^17^
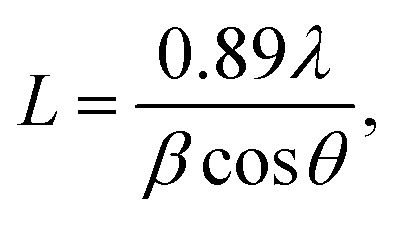
where *L* is the mean crystal size (Å) and *λ* is the wavelength of the X-ray source (1.5404 Å), thus, in the XRD pattern, *θ* and *β* correspond to the (radians) diffraction angle of the observed peaks and the full width at half maximum (FWHM), respectively. 0.89 is the Scherrer constant. Thus, for peak position, FWHM can be acquired with two Gaussian curves by fitting the measured peaks. The monochromatic Cu Kα radiation was used a X-ray source. Based on the results of X-ray diffraction, the decrease in crystallite size from (a) pure CuCr_2_O_4_ 43.12 nm, Mg-doped CuCr_2_O_4_ samples (b–d) 38.45 nm, 31.45 to 29.98 nm could be linked to maximizing the concentrations of dopant.

### 3.2. FTIR spectra

**Fig. 2:**
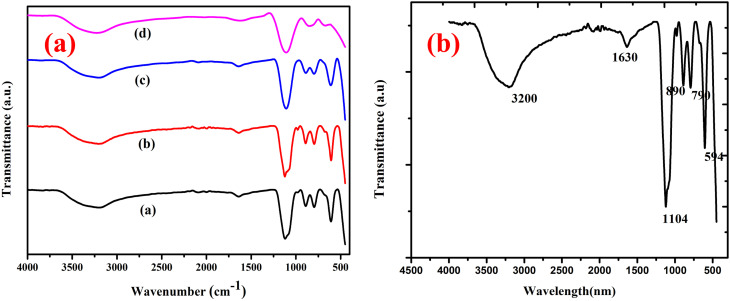
(a) FTIR spectra of (a) CuCr_2_O_4_ and (b–d) Mg-doped CuCr_2_O_4_; (b) FTIR spectra of pure CuCr_2_O_4_.

Fourier transform infrared (FTIR) spectroscopy was recorded for the four undoped samples (Fig. 2a and b) and Mg-CuCr_2_O_4_ around room temperature from 400 to 4000 cm^−1^. Thus, two sharp bands for the different absorption peaks observed around 561 and 475 cm^−1^ lower frequency band (*ν*_2_) correspond to the vibration of –Mg^2+^–Cr^3+^–O^2+^ by sites, indicating the formation of octahedrons and tetrahedrons, respectively (Table 3). The bands around 560–470 cm^−1^ correspond to the intrinsic stretching vibration of metal ions at the tetrahedral site, and the band in the range of 510–550 cm^−1^ is attributed to the M–Mg–O bond vibration. These two bands positioned a high-frequency band (*ν*_1_) at 500 and 678 cm^−1^ characteristic of CuCr_2_O_4_ spinel. The FT-IR spectra exhibited two characteristic absorption bands at 600 and 900 cm^−1^, corresponding to the tetrahedral and octahedral formations, respectively. Thus, both bands similarly correspond to the asymmetric stretching vibrations of Cr–O–Cu–Mg. In general, Mg substitution *via* transition metal ions, such as Cu^2+^, leads to a shift in the frequency bands and is due to the variation of CrO_4_ bond length.^18,19^

**Table 3:** FTIR modes (*υ*_1_ and *υ*_2_) of copper chromite and Mg-doped copper chromite

Samples	Wave number *υ*_1_ (cm^−1^)	Wave number *υ*_2_ (cm^−1^)
a	613, 801	898, 1125
b	608, 799	894, 1122
C	605, 796	890, 1120
D	661, 856	1118

### 3.3. Morphology

**Fig. 3:**
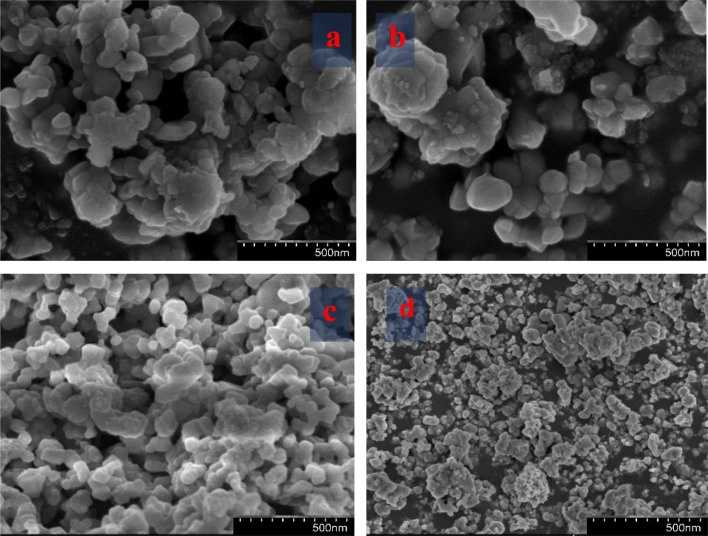
SEM showing (a) CuCr_2_O_4_ and (b–d) Mg-doped CuCr_2_O_4_.

**Fig. 4:**
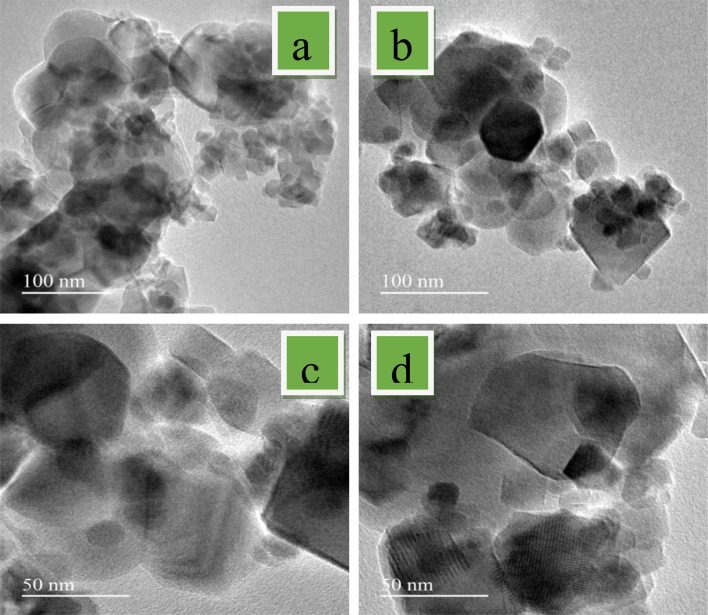
TEM images of (a) CuCr_2_O_4_ and (b–d) Mg-doped CuCr_2_O_4_.

SEM images and micrographs of the undoped and Mg-CuCr_2_O_4_ compositions (*x* = 0.00, 0.1, 0.02 and 0.03%) are demonstrated in Fig. 3a–d. The SEM images and micrographs show irregularly shaped large grains of undoped and smaller grains of Mg-CuCr_2_O_4._ In the arrangement within the SEM results, the samples have an extensive particle distribution and present strong agglomerations; hence, a measurement from the particle size was not possible. However, the obtained results give some statistical arrangements of Mg–Cu–Cr–O. As can be observed, SEM images detect particularly pure and doped sample differences between XRD crystallite sizes and particle sizes, as shown in Table 4. The crystallite size determined using the XRD approach was found to be correct, however the grain size assessed using the SEM analysis had broader values and may have measurement errors. In our studies, CuCr_2_O_4_ nanocomposites having a cluster-like structure with sizes ranging from 100 to 500 nm made by identical particles. Pure and doped samples both have discrete particle-like appropriate interfaces in CuCr_2_O_4_ nanocomposites. In addition, it is noticed that the structure can be obtained at lower synthesis temperatures from the conventional method.^20^ Thus, TEM was used to control the morphologies and particle size of copper chromite and the Mn doping copper chromite. As illustrated in Fig. 4a–d, spherical particles and regular structures are present in the doped samples. TEM images for CuCr_2_O_4_ particles were obtained and the particle-like shape changed to form spherical particles, as concentration on the Mg^2+^ is improved.

**Table 4:** Difference between the calculated crystallite sizes (XRD) of samples and the grain sizes (SEM) of copper chromite and Mg-doped copper chromites

Samples	Crystallite size from XRD (nm)	Particle size from SEM (nm)
a	43.12	49–53
b	38.45	43–39
c	31.45	40–31
D	29.98	39–29

### 3.4. Surface area analysis

**Fig. 5:**
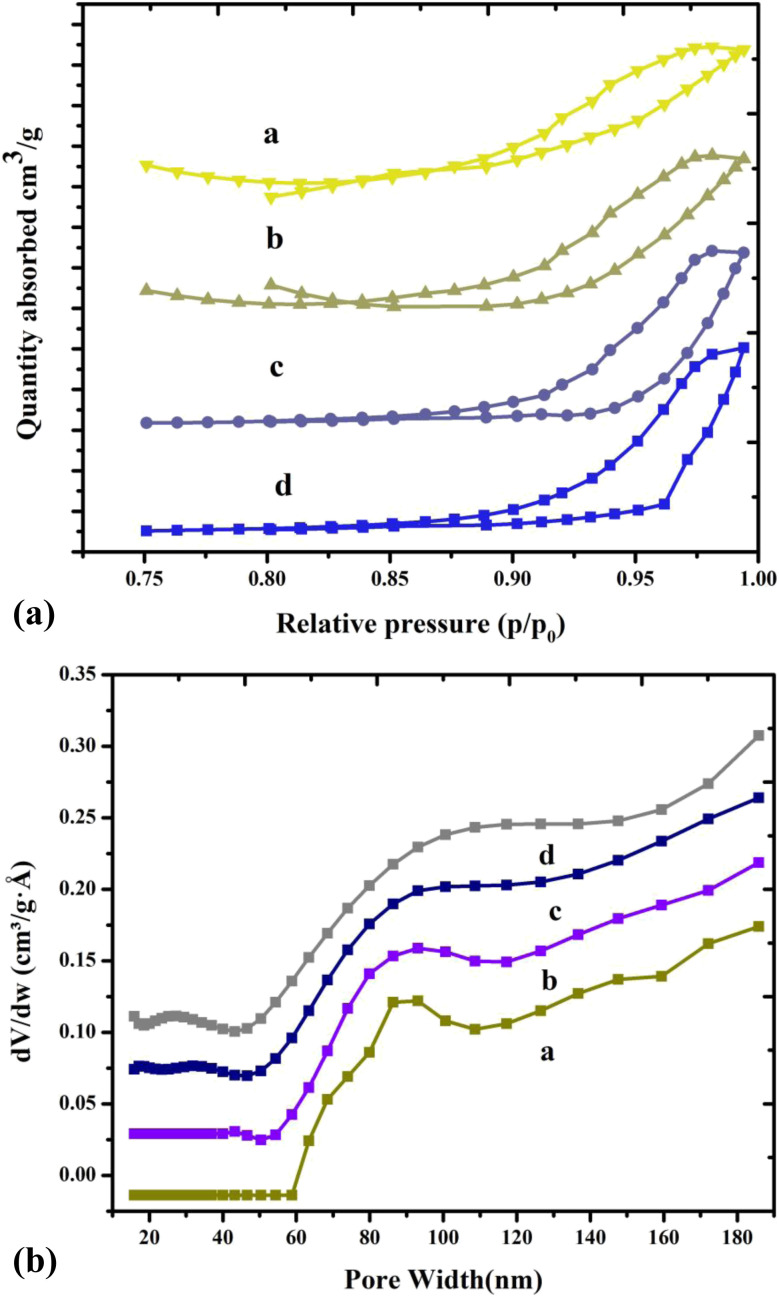
(a) N_2_ adsorption/desorption isotherms of (a) pure and (b–d) Mg-doped copper chromite. (b) Pore size distributions of (a) pure and (b–d) Mg-doped copper chromites.

Thus, a specific surface area can be used from the BET equation to determine the amount of nitrogen absorbed into copper chromite and the Mn doping copper chromite. The H–K method was used to obtain the average pore diameter, and the *t*-plot method was employed to obtain the total pore volume. In terms of catalytic activity, surface area is an important parameter when evaluating potential catalytic applications involving catalytic materials such as copper chromite and Mg-doped copper chromite. Table 5 displays the pore volume (*V*_p_), specific surface area (*S*_BET_), pore radius (*R*_p_), and crystallite size (nm). The copper chromite and Mg-doped copper chromite samples increase the surface area for converted catalytic applications because they demonstrate increasing contact between alcohol reacting molecules and aldehyde from the active sites.^21,22^ For all the samples, this was followed by the type III isotherms with the hysteresis loop at the high *P*/*P*_0_ and illuminating the representative mesoporous material.^23^ The system, copper chromite and the Mg-doped copper chromite of the plot on the amount of gas adsorbed as a function of the relative pressure are exposed altogether as a type-III (a–d) and with hysteresis loop, as shown in Fig. 5a.

**Table 5:** BET surface area, average pore diameter, pore volume and crystallite size (nm) of CuCr_2_O_4_ and Mg-doped CuCr_2_O_4_

Parameters	a	b	c	d
*S*_BET_ (m^2^ g^−1^)	38.2	40.2	57.8	63.4
*S*_mic_ (m^2^ g^−1^)	2.9	5.1	7.5	9.2
*S*_meso_ (m^2^ g^−1^)	35.3	35.1	50.0	54.1
Total pore volume (cm^3^ g^−1^)	0.1	0.1	0.1	0.2
Micro pore volume (cm^3^ g^−1^)	0.0	0.0	0.1	0.1
Meso pore volume (cm^3^ g^−1^)	0.1	0.1	0.0	0.1
Average pore diameter (nm)	13.6	14.1	17.9	19.8

When magnesium is added, the bulk copper chromite phase grows more slowly, increasing the surface area, improving porosity, and creating micropores. Because of nonisomorphic substitution, the addition of magnesium to the copper chromite phase matrix causes a decrease in grain size, which results in an increase in surface area. The three components of Mg, Cr, and Cu can form pores in their matrices, leading to the production of pores of varying sizes. The reduction in grain size and the non-isomorphic substitution of Mg in the copper chromite matrix resulted in the formation of significant micropores, which is why the average pore diameter for the Mg-doped sample decreased. Thus, the addition of Mg, which simultaneously increases the mesopores and introduces micropores, increases the catalytic activity of copper chromites.^24,25^

## 4. Catalytic activity of selective oxidation

### 4.1. Effect of temperatures

**Fig. 6:**
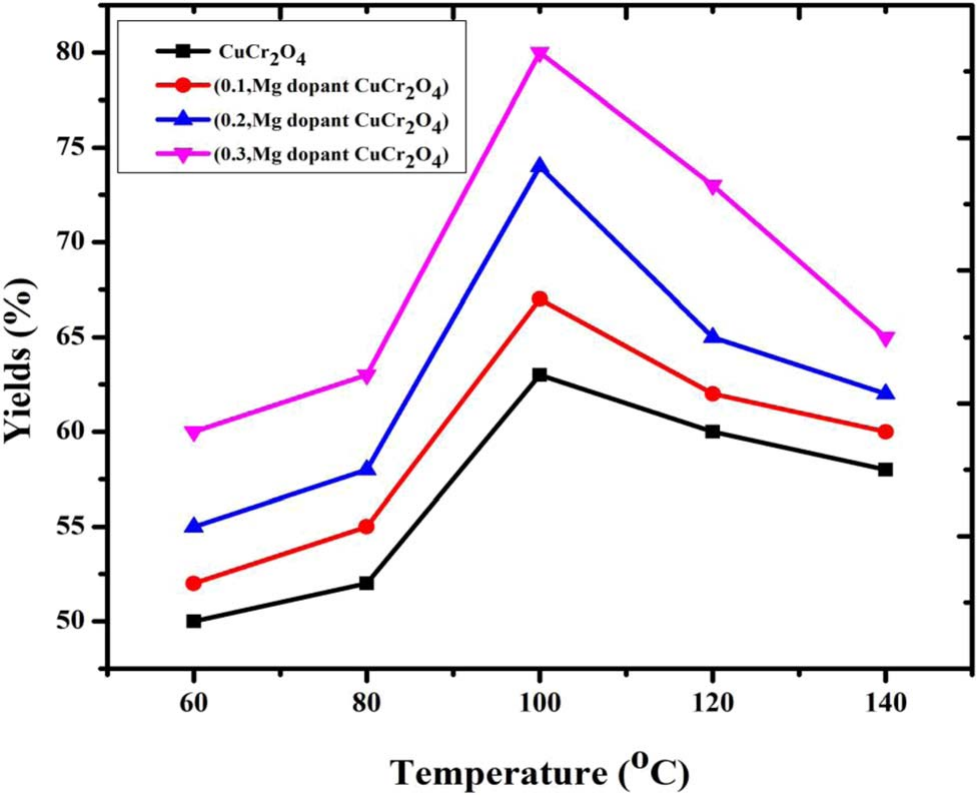
Catalytic activity of the catalysts *versus* different reaction temperatures in the oxidation of veratryl alcohol. Reaction conditions: pure and doped samples: 0.3 g; alcohol: 10 mmol; acetonitrile: 10 mmol; H_2_O_2_: 10 mmol; time: 12 h; temperature: 60 to 140 °C.

The effect of temperature on selectivity towards veratryl aldehyde and the conversion of veratryl alcohol were studied in the range of 60–140 °C, as presented in Fig. 6. The selectivity (80%) of veratryl aldehyde decreases with increasing temperature, which is particularly due to the over-oxidation of veratryl aldehyde to veratric acid. However, conversion of veratryl alcohol increased from 60 to 80% with an increase in the temperature from 60 to 140 °C.^26–28^ This system increased the reaction temperature, which was important for the decrease in the oxygen, resulting in unfavorable product formation. The optimum temperature is 100 °C.

### 4.2. Amount of catalyst

**Fig. 7:**
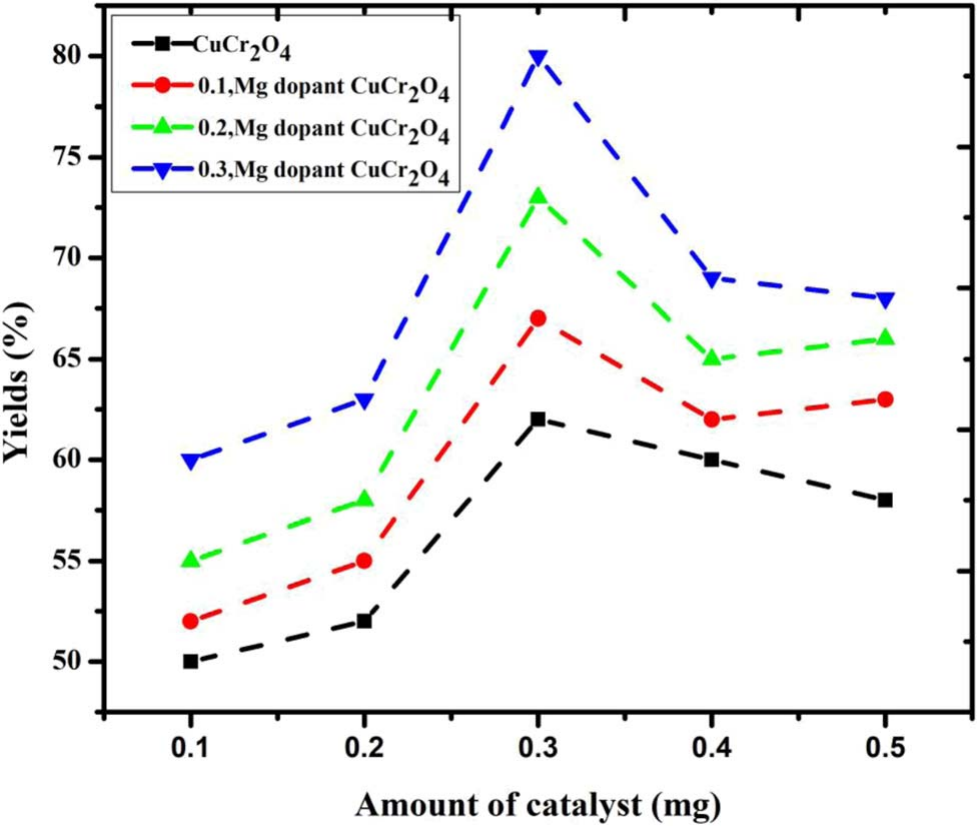
Yield of the product *via* the selective oxidation of veratryl alcohol using different catalysts. Reaction conditions: pure and doped samples: 0.1 to 0.5 g; alcohol: 10 mmol; acetonitrile: 10 mmol; H_2_O_2_: 10 mmol; time: 12 h; temperature: 100 °C.

The effect of catalyst amount (0.1 g to 0.5 g) on the selectivity of veratryl aldehyde and the conversion of veratryl alcohol into by-products formation were studied and the results are presented in Fig. 7. The catalytic activity is closely related to the following two factors: the copper chromite amount of catalyst for the active sites Cu and Cr and the size for O^2−^. The doping concentration of Mg was determined to ensure appropriate active Mg sites; however, smaller Mg particles are usually obtained. Therefore, the optimal pure and doping copper chromites are 0.3 g because they give the best yield among others. However, the amount of catalyst interaction with the catalyst regenerates the catalyst surface, causing the product molecules to desorb and encouraging additional oxidation.

### 4.3. Effect of reaction time

**Fig. 8:**
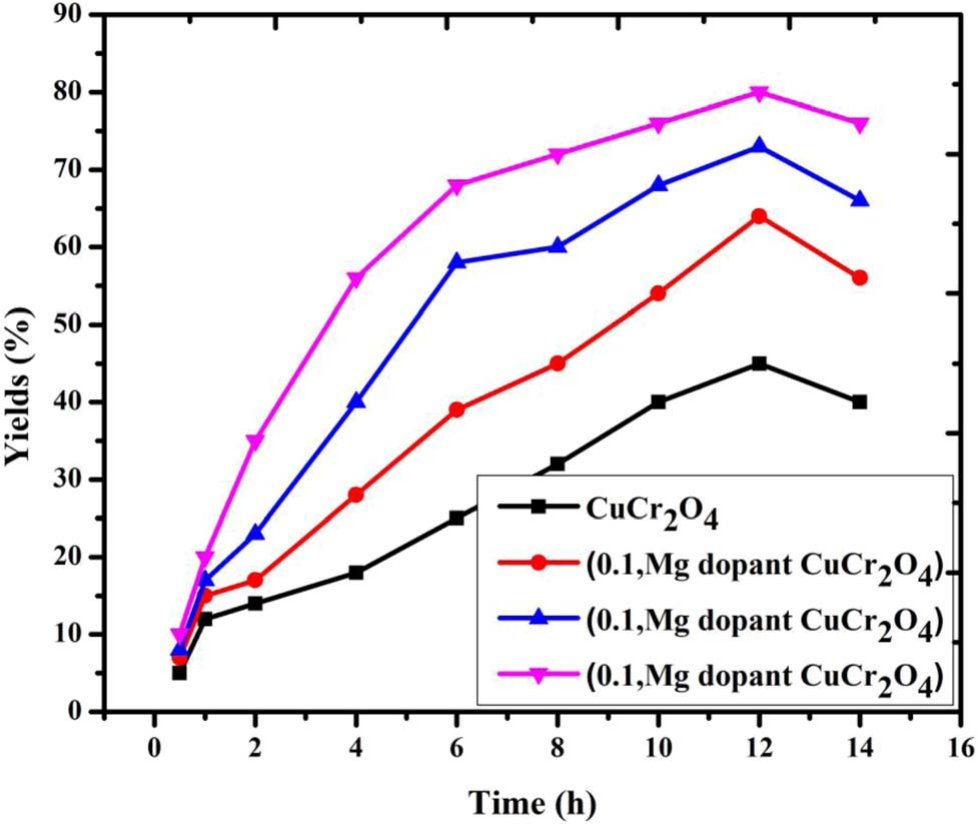
Catalytic activity of the catalysts *versus* different reaction times for the selective oxidation of veratryl alcohol. Reaction conditions: pure and doped samples: 0.3 g; alcohol: 10 mmol; acetonitrile: 10 mmol; H_2_O_2_: 10 mmol; time: 0 to 16 h; temperature: 100 °C.

This conversion of veratryl alcohol and the selective formation of product are observed at different reaction time intervals of 0.5, 1, 2, 4, 6, 8, 10, 12 and 14 h. As demonstrated in Fig. 8, 11% veratryl alcohol conversion and 50% veratryl aldehyde selectivity are obtained at 0.5 h; after allowing the reaction for 10 h, 80% yields were formed. The main product was veratryl aldehyde with veratryl acid as a by-product. Based on the catalytic reaction route, to increase the conversion of aldehyde, the reaction time needs to be prolonged. Thus, the catalytic activity was achieved with a reaction time of 12 h and a high yield was obtained, and the time was increased as the products formed.^29^ Thus, an optimum catalytic reaction time of 12 h was selected.

### 4.4. Effect of solvents

The catalytic performance was evaluated in several solvents, such as chloroform (CHCl_3_), acetonitrile (CH_3_CN) and dimethyl formamide (DMF), for the conversion of veratryl alcohol to veratryl aldehyde. Thus, a solvent plays an important role in the reaction induced by the alcohol conversion of an aldehyde decomposed for the by-products. Consequently, a higher yield for acetonitrile than for dimethyl formamide and chloroform was observed, as listed in Table 6. For this reason, a polar solvent was more easily adsorbed on the catalyst surface, preventing the adsorption of alcohol to aldehyde. Because the substrates and oxidants are more soluble in the solvent, they can more readily bind to the catalyst's active sites. Therefore, acetonitrile was the more active solvent for the catalytic system under study. In the case of CHCl_3_ and DMF, the yields are lower compared to acetonitrile, might be due to the inefficient hydrogen transfer by solvents, and the incomplete solubility of organic substrates and catalytic systems in the solvents. It turned out that the conversion of alcohol depended on the polarity of the solvent molecules, polarity order from acetonitrile, dimethyl formamide and chloroform. Besides, acetonitrile was selected as the solvent for the catalytic system.

**Table 6:** Yield of the product in different oxidants obtained *via* veratryl alcohol^a^

Samples	Chloroform	Dimethyl formamide	Acetonitrile
a	32	40	46
b	45	54	65
c	59	67	78
d	68	73	83

a Reaction conditions: pure and doped samples: 0.3 g; alcohol: 10 mmol; acetonitrile: CHCl_3_ and DMF 10 mmol; H_2_O_2_: 10 mmol; time 12 h: temperature: 100 °C.

### 4.5. Effect of oxidants

**Fig. 9:**
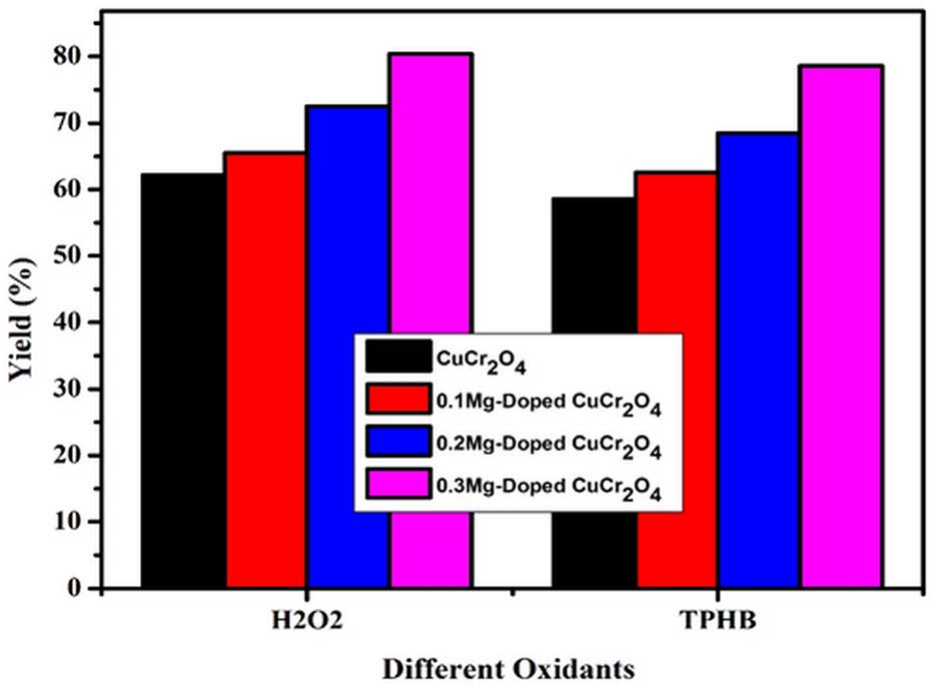
Effect of oxidants in the selective oxidation of veratryl alcohol. Reaction conditions: pure and doped samples: 0.3 g; alcohol: 10 mmol; acetonitrile: 10 mmol; TBHP and H_2_O_2_: 10 mmol; time: 12 h; temperature: 100 °C.

Catalytic performance for oxidation as a function of the oxidant played an important role. This catalytic reaction is controlled by the option for the reaction to occur due to the contribution of the oxygen lattice (Fig. 9) from the oxidant. Hereafter, the reaction was successively carried out in the presence of different oxidants, such as TBHP and hydrogen peroxide. TBHP was slightly soluble on the selected organic substrate and the catalytic system. As a result, the yield of veratryl aldehyde is low in all synthesized CuCr_2_O_4_ nanomaterials. In the case of H_2_O_2_, the solubility on the chosen organic substrate could be higher. In conclusion, H_2_O_2_ on acetonitrile was usual because the establishment of the peroxycarboximidic acid was intermediate and the transfer agent was good oxygen. Hence, the choice of the oxidant was hydrogen peroxide.^30,31^

### 4.6. Mechanism

**Scheme 1:**
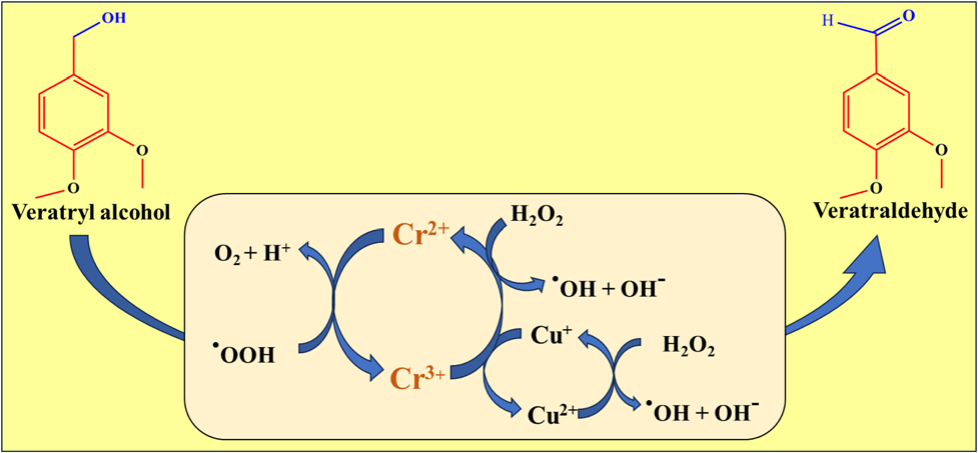
Shows the selective oxidation of veratryl alcohol to veratraldehyde.

A proposed mechanism in Scheme 1 suggests that H_2_O_2_ molecules undergo homolytic cleavage, resulting in two hydroperoxy radicals. These radicals react with Cr^3+^ and Cu^2+^ ions on the surface of CuCr_2_O_4_, creating Cr^2+^ and Cu^+^ ions and regenerating hydroperoxyl. The hydroperoxy radicals also react with hydroxy radicals on the surface to form water and oxygen. Veratryl alcohol molecules are adsorbed onto the surface of CuCr_2_O_4_, forming metal-alkoxide intermediates and adsorbing hydrogen *via* O–H bond cleavage. The surface O–H group is formed by bonding adsorbed hydrogen with the surface lattice oxygen and coordinating with the carbon atom of the methyl group in the veratryl alcohol molecule. Finally, the C–H bond in the *β* position is activated, resulting in the formation of veratraldehyde and water. The oxygen adsorbed onto the support surface dissociates into chemisorbed oxygen species and diffuses into the lattice vacancies of the support. This is followed by activation in the oxygen vacancies and conversion into lattice oxygen (O^2−^). The lattice oxygen further transfers to the surface of CuCr_2_O_4_ through oxygen-deficient sites, restoring the interface between the CuCr_2_O_4_ active sites.^32–35^

1 OH^−^ + H_2_O_2_ ↔ HO_2_^−^ + H_2_O

2 2H_2_O_2_ + Cr^2+^ + Cu^+^ ↔ Cr^3+^ + Cu^2+^ + 2OH˙ + 2OH^−^

3 2HO_2_^−^ + Cr^3+^ + Cu^2+^ ↔ Cr^2+^ + Cu^+^ + 2HO_2_˙

4 HO_2_˙ + OH˙ ↔ H_2_O + O_2_

### 4.7. Recyclability of the catalyst

**Fig. 10:**
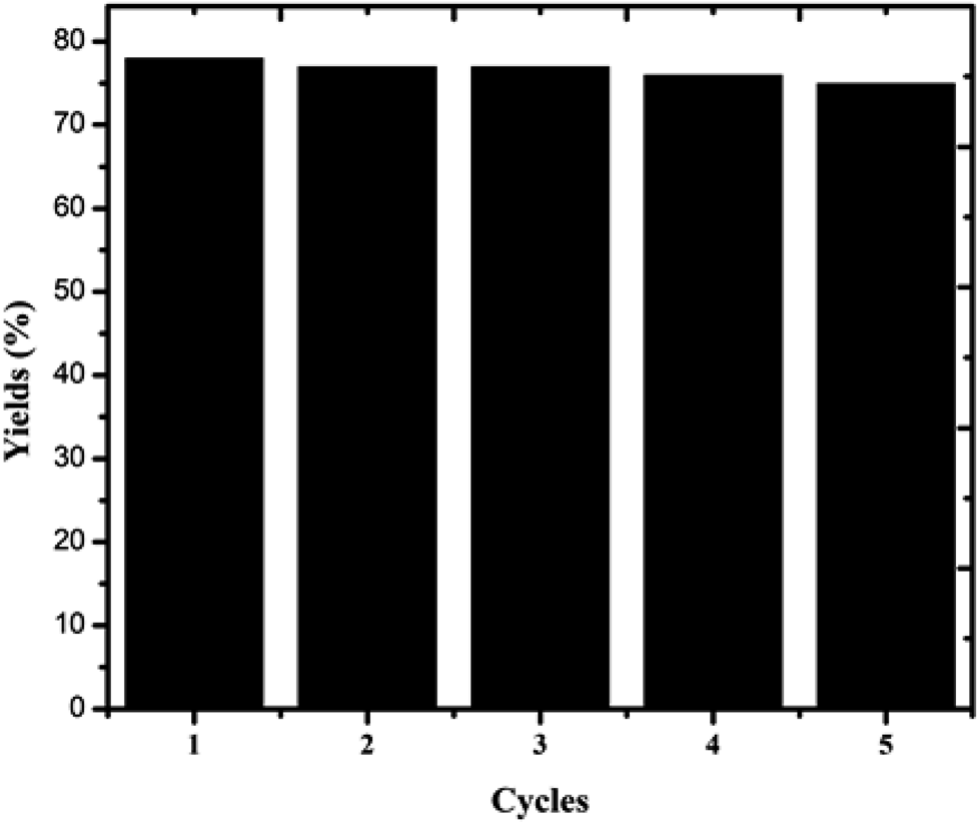
Cycling performance of the copper chromite catalysts.

To examine the recyclability of the catalyst, the oxidation of veratryl alcohol to veratraldehyde was repeated five times with the same catalyst. The percentage conversion of veratraldehyde slightly decreased. The catalysts after the reaction were recovered by centrifugation, washed with ethanol, and then dried and reused for another reaction under the same conditions, as shown in Fig. 10. From this determination, the samples were filtered and the residue was washed several times with acetone and dried at 120 °C in an air oven for 3 h; this was repeated five times under identical catalytic conditions. Consequently, considering the small amount of catalyst used in the reaction, the great loss of catalyst should be due to the process of washing, which could be easily addressed in the large scale industrial production.

During the catalytic oxidation of veratryl alcohol to veratraldehyde, O–H bonds are broken and attended through the formation of hydrogen atoms, which further react with oxygen to generate water to achieve dehydrogenation to produce veratraldehyde. Subsequently, one hydrogen atom in the CHO groups was formed *via* a broken O–H bond, forming a metal–alcohol salt intermediate. Further, the objectivity of the hydrogen atom from carbon induces the conversion of the alcohol group into an aldehyde.

## 5. Conclusions

This paper demonstrates a simple method for producing efficient catalysts for the catalytic oxidation of veratryl alcohol to veratraldehyde. The further detachment of a hydrogen atom from carbon causes the alcohol group to be converted into an aldehyde group, resulting in the creation of an intermediate and veratric acid. XRD confirmed the development of the spinel phase. Thus, pure and Mg-doped spinels were found to be highly porous, as confirmed by SEM. Thus, copper chromite nanocomposites with a crystallite size in the range of 43–29 nm were synthesized. FT-IR was used to confirm the formation of the spinel phase. The current technology is applicable to various d and f block metal-supported catalysts for further catalytic processes.

## Conflicts of interest

There are no conflicts to declare.

## Supplementary Material
